# Activation of cGAS-STING Signal to Inhibit the Proliferation of Bladder Cancer: The Immune Effect of Cisplatin

**DOI:** 10.3390/cells11193011

**Published:** 2022-09-27

**Authors:** Guanghou Fu, Yunfei Wu, Guanan Zhao, Xiaoyi Chen, Zhijie Xu, Junjie Sun, Junjie Tian, Zhengjun Cheng, Yue Shi, Baiye Jin

**Affiliations:** Department of Urology, The First Affiliated Hospital, Zhejiang University School of Medicine, Hangzhou 310003, China

**Keywords:** bladder cancer, cisplatin, cGAS-STING, DNA damage, chromatin bound

## Abstract

Cisplatin is commonly used in neoadjuvant, adjuvant, and systemic therapy for advanced bladder cancer, but its immune-related mechanism is still unclear. Exploration of the immune effects of cisplatin in bladder cancer would complement the comprehensive mechanism of cisplatin and provide the basis for combination therapy of cisplatin and immunotherapy in bladder cancer. We confirmed the immune effects of cisplatin on T24 and TCCSUP bladder cancer cell lines in vitro and explored the important function of these immune effects in the bladder cancer microenvironment in a mice tumor model. We found cisplatin induced immune response in bladder cancer by RNA sequencing and validated that cGAS-STING signal was deeply involved in this response. Cisplatin induced cGAS-STING signal inhibited the proliferation of bladder cancer and increased the infiltration percentages of CD8+ T cells and dendritic cells in a transplantation mice tumor model. Accumulation of dsDNA and the release of chromatin bound cGAS are important to activate downstream STING. Our findings indicated a cisplatin-related immune effect in bladder cancer, and cisplatin combined with immunotherapy might have a synergistic effect for bladder cancer therapy.

## 1. Introduction

Bladder cancer is one of the most common tumors in the urinary system [1]. Based on pathology, bladder cancer is mainly divided into NMIBC (non-muscle invasive bladder cancer) or MIBC (muscle invasive bladder cancer), with the latter demonstrating a poorer prognosis. Thus, MIBC is recommended as an aggressive treatment, such as cystectomy combined with chemotherapy. More than 80% of bladder cancer chemotherapy is based on platinum drugs, and cisplatin is the most classic drug among them [2]. However, more than 60% of patients receiving chemotherapy strategies, such as neo-adjuvant chemotherapy, are cisplatin-resistant [3]. The critical mechanism of cisplatin resistance is still unclear. As a classical DNA cross linker, intracellular cisplatin can inhibit tumor cells by inducing irreversible structural distortion of DNA, which causes DNA damage and results in accumulated production of dsDNA or micronuclei in tumor cells [4,5]. More and more studies suggested that cisplatin can also indirectly affect the immune cell components in the tumor microenvironment, which is closely related to tumor recurrence, progression, and drug resistance [6,7].

The cGAS-STING pathway has been found to be involved in many disease processes, such as immune defense, tumor progression, autoimmune diseases, and neuron degeneration [8,9,10,11,12]. It has been reported that cGAS, an intracellular dsDNA (double-stranded DNA) receptor, can recognize and catalyze cytosolic dsDNA induced by cisplatin, thus activating the ownstream STING signal [4]. Meanwhile, some studies found that cGAS and STING protein are indispensable for the antitumor effect of immune checkpoint blockade [13,14,15], which provided new sights for the combination of chemotherapy with immunotherapy. After recognition of double-stranded DNA (dsDNA), cGAS catalyzes the cyclization of ATP and GTP into the second messenger cyclic GMP–AMP (2,3-cGAMP). cGAMP activates STING and results in the higher-ordered tetramerization of STING and translocation of STING from the endoplasmic reticulum to golgi compartments, where STING is palmitoylated and serves as a signaling platform for recruiting and activating TBK1 and IKK. Subsequently, TBK1 recruits and phosphorylates IRF3, which leads to translocation of IRF3 to the nucleus. IKK-mediated phosphorylation of the inhibitory IκB protein permits nuclear entry of nuclear factor κB (NF-κB). Finally, NF-κB with IRF3 in the nucleus leads to the secretion of type I interferons, such as IFN-β [4,16].

The functional activity of cGAS is the critical step in activating the STING pathway. Liu et al. found that the nuclear translocation of cGAS in THP-1 and HeLa cells can affect its functions by suppressing homologous recombination-mediated repair [17]. Studies have also shown that the binding capacity of cGAS to nucleosomes is much greater than that of cGAS to dsDNA [18,19,20,21,22], which can explain why the cGAS-STING signal is not significantly activated during mitosis when a large amount of dsDNA is exposed to the cytoplasm due to the dissolved nuclear membrane. Since cGAS is competitively bound by nucleosomes, it may lose the ability to bind dsDNA and activate STING downstream [18,19,20,21,22].

We found that cisplatin treatment induces cGAS-STING-dependent immune responses in bladder cancers. Cisplatin-related immune responses promote the secretion of cytokines such as IFN-β and IL-6 in vitro and can recruit CD8+ T cells and dendritic cell (DC) infiltration in a mouse tumor microenvironment. Cisplatin-induced DNA damage can activate intracellular cGAS by producing dsDNA fragments and micronuclei. It can also dissociate cGAS from chromatin by distorting the structure of nucleosomes, restore the ability of cGAS to bind dsDNA and then activate STING signaling. Exploration of the immune effects of cisplatin in bladder cancer can provide a better understanding of the diverse mechanisms of cisplatin and determined the basis for combining cisplatin and immunotherapy.

## 2. Materials and Methods

### 2.1. Cell Preparation and Culture

Human-derived MIBC cell lines T24 (RRID: CVCL_0554), TCCSUP (RRID: CVCL_1738), and UMUC-3 (CVCL_1783), mouse derived bladder cell lines MB49 (RRID: CVCL_7076), and 293T (RRID: CVCL_0063) cell lines were purchased from the Cell Bank of the Chinese Academy of Sciences (Shanghai, China). All human cell lines have been authenticated using short tandem repeat DNA profiling analysis with the last three years to ensure the stable and reliable of all cell lines during the experiments. T24, TCCSUP, UMUC-3 and MB49 cells were cultured in RPMI-1640 medium (Thermo Fisher Scientific, Waltham, MA, USA.), and 293T cells were cultured in DMEM medium (Thermo Fisher Scientific, Waltham, MA, USA). Mediums mentioned before were pre-supplemented with 10% fetal bovine serum (Gibco; Thermo Fisher Scientific, Waltham, MA, USA.) without any antibiotics, and all cell lines were incubated under standard conditions (37 °C and 5% CO_2_). Moreover, all experiments were performed with mycoplasma-free cells. According to our previous research and related literature [23,24], the IC50 of human bladder tumor cell lines against cisplatin was between 1.997 μg/mL and 2.585 μg/mL. Therefore, the concentration of 2 μg/mL cisplatin was selected for the further experiments.

### 2.2. Cell Counting Kit-8 (CCK-8) Assay and Colony Formation Assay

Each group of T24, TCCSUP, and MB49 cell lines were prepared in 96-well plates (1000 cells/well) under standard conditions. Then, premixed medium with a 10% concentration of CCK-8 (Dojindo Molecular Technologies, Rockville, MD, USA) reagent was added into each well followed by incubating under standard conditions for 2 h before measurement at 450 nm. For colony formation assay, different groups of pretreated cells were prepared in 6-well plates (1000 cells/well) and cultured for 7 days under standard conditions (37 °C and 5% CO_2_). On the last day, 6-well plates were firstly washed by PBS for 3 times, successively following 3 min solution A staining, 6 min solution B fixation, and 3 times of PBS washing (Wright-Giemsa Stain Kit, NJJC bioengineering institution, Nanjing, China). Finally, the differences of colony formation assay were analyzed by Image J (version 1.8.0).

### 2.3. Plasmids Construction and Transfection

Sequences of short hairpin RNA (shRNA) against CDS of msSTING were validated from Sigma-Aldrich Online. Scramble sequences were designed using Wizard v3.1 to ensure the absence of seed sequence matches. msSTING sequences and scramble sequences were synthesized (Tsingke, Hangzhou, China) and inserted into the plko.1-puro vector. Then, the vector was co-transfected with pSPAX2 and pMD2G (with a ratio of 4:3:1, respectively) into the 293T cell lines cultured in 100 mm plates (30–60% cell density) using Lipofectamine 3000 (Thermo Fisher Scientific, Waltham, MA, USA). The supernatant, which contained Lentivirus, was harvested and centrifuged (800× *g*, 5 min) 48 or 72 h post-transfection and added into MB49 cells cultured in 6-well plates (30–60% cell density) immediately. Successfully transfected shSTING cell lines were screened with an increasing concentration of puromycin (Sigma-Aldrich; Merck KGaA, Darmstadt, Germany). The siRNA used here were also transfected using Lipofectamine 3000. Finally, the transfection efficiency was validated by western blotting. The msSTING and siSTING sequences used here were showed in Table S1.

### 2.4. Mice

C57BL/6 mice were purchased from Jackson Labs. Four-week-old mice were mainly used in this experiment, and all mice were maintained in pathogen-free barrier facilities and were approved by Zhejiang University experimental animal welfare ethics review committee. Before the experiment, 24 mice were selected and randomly divided into 4 groups with 6 mice in each group: A. PBS+shSCR group; B. PBS+shSTING group; C. Cisplatin+shSCR group; D. Cisplatin+shSTING group. 2 × 10^6^ of MB49 cells with shSTING or shSCR transfection were subcutaneously injected on the flank of mice. Then, cisplatin (3 mg/kg) or the same volume of PBS were injected intratumorally in day 13 and day 16. Tumor volume was measured in day 10, 13, 16, 19, and 22 and calculated as 1/2 × length (L) × width (W) × height (H). Then, the tumors were harvested in day 23, the weight of tumors were normalized by their corresponding body weight. Fresh tumor issues were then used for flow cytometry analysis.

### 2.5. RNA-Sequence (RNA-seq) and Reverse Transcription-Quantitative Polymerase Chain Reaction (RT-qPCR)

Trizol (Thermo Fisher Scientific, Waltham, MA, USA) was used for cells total RNA extraction according to manufacturer’s protocol. Then, different groups of RNA were used for RNA-seq or qRT-PCR. The sequencing coverage and quality statistics for each sample are summarized in Supplementary Table S2. T24 cells were treated with cisplatin (2 μg/mL) or PBS for 24 h under standard condition. RNA-sequence was performed by Novogene China. Gene expression levels were quantified as Fragments Per Kilobase Million and then Log_2_ –transformed as A value. The significantly different expression genes were identified as absolute fold change of A value > 1.5 combined Padj (adjust *p*) value < 0.05 between two groups. Then, different expression genes were used for plotting heatmap and volcano map by R soltware (Version 4.0.2, https://cran.r-project.org/src/base/R-4/, accessed on 8 October 2020). GO and KEGG analysis were performed on DAVID online (https://david.ncifcrf.gov/, accessed on 9 October 2020). Pathways with Padj value < 0.05 were identified as significant different pathway. As for qRT-PCR, Takara PrimeScript™ RT and SYBR EX Taq™ kits (Takara, Tokyo, Japan) were used for qRT-PCR according to manufacturer’s instruction. The specific condition used was as follows: Initiate Step, 95.0 °C: 30 s; cycling Step, 40 cycles of 95 °C: 5 s and 60 °C: 30 s; melt curve analysis Step, 65 °C to 95 °C, increasing in 0.5 °C increments for 5 s. The specific primers used in this experiment were listed in Table S1. Control groups were conducted to confirm the absence of the agent pollution or primer dimers. GAPDH expression was chosen as the control, and the relative mRNA expressions of targeting genes were calculated using the ΔΔCq method.

### 2.6. Enzyme Linked Immunosorbent Assay (ELISA) and IFN Reporter Assay

Secreted supernatant IL-6 of T24 and TCCSUP cell lines was measured using the Human IL-6 ELISA kit (DAKEWE, Shenzhen, China) according to manufacturer’s instructions. IFN-β in cell supernatants with biological activity was compared using a reporter 293T cells stably expressing a pISRE (Genechem, Shanghai, China). Reporter cells were co-cultured with supernatant from different groups for 24 h before being measured by a fluorescent enzyme meter Varioskan™ Flash at 488 nm/520 nm (Thermo Fisher Scientific, Waltham, MA, USA). Intensities of cellular fluorescent were correcting by the total number of T24 and TCCSUP cells.

### 2.7. Subcellular Fractionation and Western Blotting Assay

Subcellular fractionation protein was extracted by Subcellular Protein Fractionation Kit for Cultured Cells (Thermo Fisher Scientific, Waltham, MA, USA) and NE-PER™ Nuclear and Cytoplasmic Extraction Reagents (Thermo Fisher Scientific, Waltham, MA, USA) according to the kit instruction. As for total protein extraction, T24, TCCSUP and MB49 cells were washed using cold PBS before being lysed using RIPA Lysis buffer with 1% cocktail protease inhibitor (Fdbio Science, Hangzhou, China). After ultrasonic spallation at 4 °C, the protein samples were centrifuged (4 °C, 15,000× *g*, 15 min) and the clear supernatant extracts were quantification using BCA kit (Thermo Fisher) and 15–25 µg/10 µL denatured protein samples were prepared and loaded in 4–12% Tris-acetate gels (Genscript, Shanghai, China) and then separated by electrophoresis. Then, the proteins were transferred onto a polyvinylidene fluoride membrane with 0.45 μm pore size. Then, the membrane was blocked with 5% bovine serum albumin (Fdbio Science, Hangzhou, China) in Tris-buffered saline containing 1% Tween-20 (TBST) for 1 h at room temperature and further incubated with primary antibodies for 12 h in a shaker at 4 °C. Subsequent to washing with TBST 5 min for three times, the membrane was incubated with secondary antibodies for 1 h at room temperature. Specific information of primary antibodies: Human-Reactive STING Pathway Antibody Sampler Kit (1:1000; cat no. #38866;CST, Danvers, MA, USA), Mouse-Reactive STING Pathway Antibody Sampler Kit (1:1000; cat no. #16029; CST, Danvers, MA, USA), γ-H2A.X (1:1000; cat no. ab81299; Abcam, Cambridge, England), Rad51(1:1000; cat no. ab133534; Abcam), GAPDH (1:5000; cat no. ab8245; Abcam), and Histone H3 (1:1000; cat no. ab1791; Abcam). The information of abovementioned secondary antibodies: Goat anti-Rabbit-HRP (1: 3000; cat no. PDR007; Fdbio Science, Hangzhou, China) and Goat anti-Mouse-HRP (1: 3000; cat no. PDM007; Fdbio Science, Hangzhou, China). The blotting results were illustrated by Dura ECL detection kit (Fdbio Science, Hangzhou, China), and specific protein bands were analyzed using Image-Pro Plus software 6.0 (Media Cybernetics, Rockville, MD, USA). GAPDH was selected as a total control protein or cytosolic control protein, histone H3 was selected as total nuclear control protein or chromatin-bound control protein.

### 2.8. Flow Cytometry Analysis

Tumor tissues were digested and incubated with biotinylated anti-CD3, anti-CD8 antibodies, followed by incubated with streptavidin-PE/Cy7 (Cat no.SA1012; Invitrogen™; Thermo Fisher Scientific, Waltham, MA, USA), anti-CD45.2-APC for 10 min at room temperature in darkroom. To analyze DCs, tissue cells were incubated with anti-MHCⅡ-APC and anti-CD11c-PE for 10 min at room temperature in a darkroom. To analyze macrophage cells, tumor cells was collected and stained with anti-CD11b-APC and anti-F4/80-PE for 10 min at room temperature in a darkroom. Specific antibodies used in flow cytometry analysis were: anti-CD3 (1:200; cat no. 13-0032-82; eBioscience™; Thermo Fisher Scientific, Waltham, MA, USA), anti-CD8 (1:200; cat no. 13-0081-82; eBioscience™; Thermo Fisher Scientific, Waltham, MA, USA), anti-CD45.2-APC (1:200; cat no. 17-0454-82; eBioscience™; Thermo Fisher Scientific, Waltham, MA, USA), anti-MHCⅡ-APC (1:200; cat no. 17-5321-82; eBioscience™; Thermo Fisher Scientific, Waltham, MA, USA), anti-CD11c-PE (1:200; cat no. 12-0114-82; eBioscience™; Thermo Fisher Scientific, Waltham, MA, USA), anti-CD11b-APC (1:200; cat no. 17-0112-82; eBioscience™; Thermo Fisher Scientific, Waltham, MA, USA), and anti-F4/80-PE (1:200; cat no. 17-4801-82; eBioscience™, Waltham, MA, USA). Then, the infiltration percentages of different cell types in tumors were calculated by BD FACSCanto™ II (BD Biosciences, San Jose, CA, USA) and then analyzed by FlowJo 10.0 (FlowJo LLC, Ashland, OR, USA).

### 2.9. Immunofluorescence

Different groups of T24, TCCSUP, UMUC-3, and MB49 cells were pre-seeded the day before immunofluorescence assay. For cell fixation, 4% paraformaldehyde was used, and 0.5% Triton-X 100 was used to increase cell membrane permeability for 10–15 min at room temperature. Then, 3–5% BSA was used for 30 min at room temperature to block antibodies. Cells were further incubated with primary antibodies overnight at 4 °C and subsequently incubated with corresponding fluorescence antibodies combined with DAPI staining 60 min in a darkroom and then washed twice by PBS before being observed by NIS A1 laser confocal microscope (Nikon, Tokyo, Japan). The primary antibodies used for immunofluorescence assay were: cGAS (1:200; cat no. A8335; Abclonal, Wuhan, China), F-actin (1:1000; cat no. A12380; Invitrogen™; Thermo Fisher Scientific, Waltham, MA, USA), Rad51(1:300; cat no. ab133534; Abcam, Cambridge, England), γ-H2A.X (1:300; cat no. ab81299; Abcam, Cambridge, England). The secondary antibodies used for immunofluorescence assay were: Goat Anti-Rabbit Alexa Fluor 488 (1:200; cat no. ab150077; Abcam, Cambridge, England), and Goat Anti-Mouse Alexa Fluor 594 (1:200; cat no. ab150116; Abcam, Cambridge, England). The results were analyzed by NIS-Elements Viewer 4.50 (Nikon, Tokyo, Japan).

### 2.10. Statistical Analysis

SPSS 22.0 (IBM, Armonk, NY, USA) was used to normalize and analyze raw data in this experiment. Data was presented as the mean ± standard deviation. Kaplan–Meier survival method and log-rank test analysis were using to plot (OS) overall survival and (DFS) disease-free survival rates curves. A Student’s *t*-test was used to assess the different between two groups. One-way ANOVA test was performed to assess the differences in multiples groups followed by Student–Newman–Keuls post hoc test. IC50 of MB49 cell lines were measured by Probit regression analysis. *p* < 0.05 was considered to have statistical significance. Data in experiments were repeated at least 3 times.

## 3. Results

### 3.1. Cisplatin Induces Specific Immune Effects in Bladder Cancer Cell Lines

**Figure 1:**
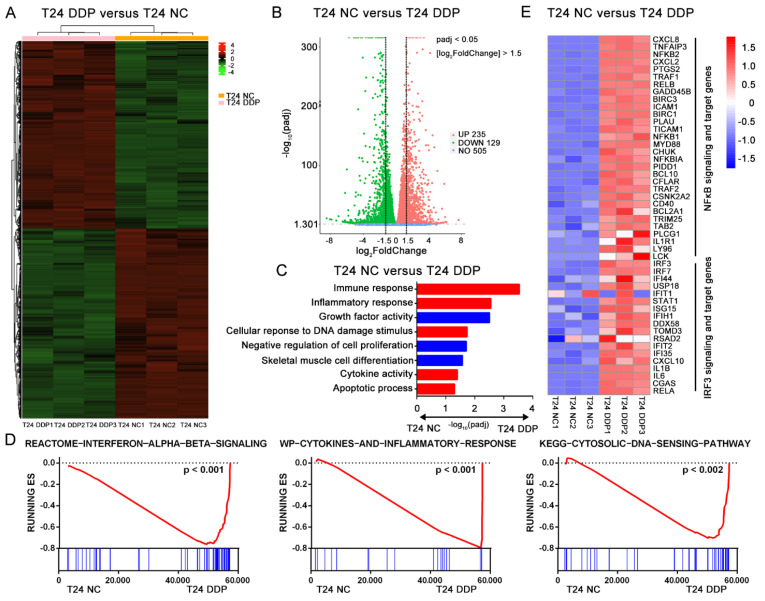
Cisplatin induces specific immune effects in bladder cancer cell line. T24 cell lines were treated with 2 μg/mL cisplatin (T24 DDP group) or PBS (T24 PBS group) for 24 h at standard condition. (**A**) The different genes expression panels between T24 DDP group and T24 PBS group were showed in heatmap. (**B**) Gene expression levels were quantified as Fragments Per Kilobase Million (FPKM) and then Log_2_–transformed as A value. The significant different expression genes were identified as absolute fold change of A value > 1.5 and Padj (adjust *p*) value < 0.05 between two groups. Finally, the volcano map showed 235 up genes and 129 down genes with significant difference. (**C**) Go pathways with Padj value < 0.05 were showed here between T24 DDP group and T24 PBS group. (**D**) Gene set enrichment analysis (GSEA) in: Interferon α, β signaling (*p* < 0.001), cytokines and inflammatory response (*p* < 0.001) and cytosolic DNA sensing pathway (*p* < 0.002). (**E**) Relative transcription levels of genes expression involved in IRF3 and NF-κB signal pathway or targeting genes in T24 DDP group and T24 PBS group. Gene expressions were normalized by R software from −1.5 to 1.5 (Low to high, blue to red).

To explore the specific effect of cisplatin on bladder cancer, the MIBC-derived cell line T24 was treated with cisplatin (2 μg/mL, 24 h) followed by RNA Seq. We found a significant difference in gene expression between the cisplatin-treated groups and the control groups (Figure 1A). The combination of absolute value log_2_ (Fold Change) > 1.5 and Padj (adjusted *p*) < 0.05 was chosen as the criteria for screening significant differential genes between the two groups, and we ultimately obtained 235 upregulated and 129 downregulated genes (Figure 1B). After the GO and KEGG (https://david.ncifcrf.gov/, accessed on 9 October 2020 ) analyses of these differentially expressed genes, we found that the cisplatin treatment mainly affected T24 cells in immune response, inflammatory response, cytokine activity, cellular response to DNA damage stimulus, apoptosis process, and some cell growth-related pathways (Figure 1C). A gene set enrichment analysis (GSEA) also confirmed the strong enrichment in immune- and inflammation-related pathways among the top 20 differentially expressed pathways in the cisplatin-treated group. Interestingly, we noticed that the type I interferon-related pathway and cytosolic DNA sensor pathway were significantly upregulated after cisplatin stimulation (Figure 1D and Figure S1A,B). The cellular response to the cytosolic DNA sensor process is closely related to the cGAS-STING pathway. Recently, it has been reported that cGAS, an intracellular dsDNA receptor, can recognize and catalyze dsDNA induced by cisplatin, thus activating the downstream STING pathway [4]. Then, we explored the activation of downstream cGAS-STING and found that the transcription levels of NF-κB- and IRF3-related genes were significantly upregulated after the cisplatin treatment (Figure 1E). These results suggest that the immune effects associated with cisplatin are due to the activation of the cGAS-STING pathway [4,8]. 

### 3.2. Immune Effect of Cisplatin in Bladder Cancer Is Related to the Activation of the cGAS-STING Pathway

**Figure 2:**
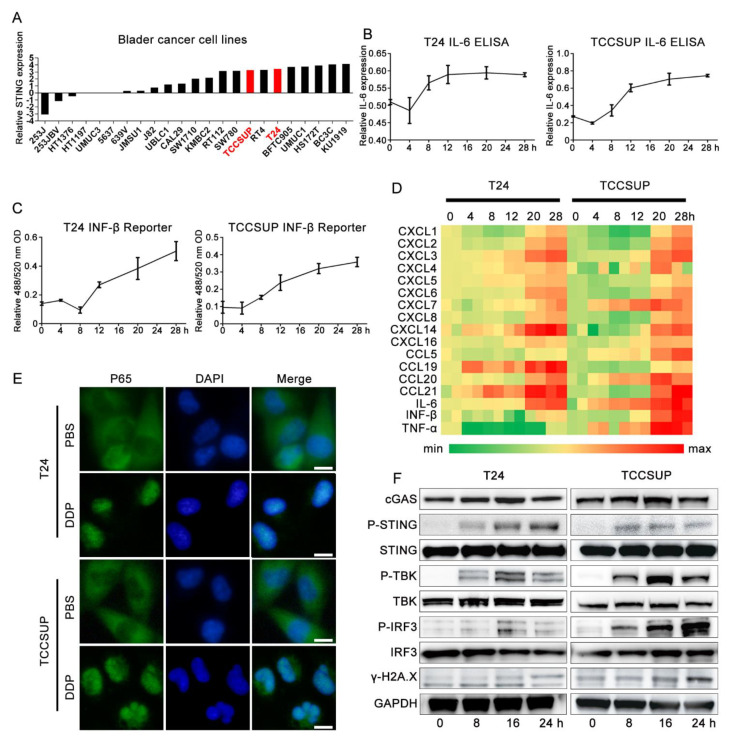
Immune effect of cisplatin in bladder cancer is related to the activation of cGASSTING pathway. (**A**) Basic transcription levels of STING in bladder cancer cell line on CCLE database. (**B**–**D**) T24 and TCCSUP cell lines were treated with 2 μg/mL cisplatin for 0 h (control group), 4 h, 8 h, 12 h, 20 h, 28 h at standard condition, respectively. (**B**) ELISA analysis of IL-6 secretion in supernatants in cisplatin treated T24 and TCCSUP cell lines and corresponding control groups for different time points. (**C**) IFN Bio-assay in cisplatin treated T24 and TCCSUP cell lines and corresponding control groups for different time points. (**D**) qRT-PCR analysis of cytokines and chemokines expression in cisplatin treated T24 and TCCSUP cell lines and corresponding control groups for different time points. Shown are genes induced at least two repeats. (**E**) Immunofluorescence assay showed subcellular distribution of p65 in T24 and TCCSUP cell lines treated with 2 μg/mL cisplatin or PBS for 24 h. Scale bar represent 5 μm. (**F**) Western blotting results of key proteins involved in cGAS-STING pathway in T24 and TCCSUP cell lines treated with 2 μg/mL cisplatin for 0 h, 4 h, 8 h, 12 h, 24 h, respectively. GAPDH was used as control protein.

**Figure 3:**
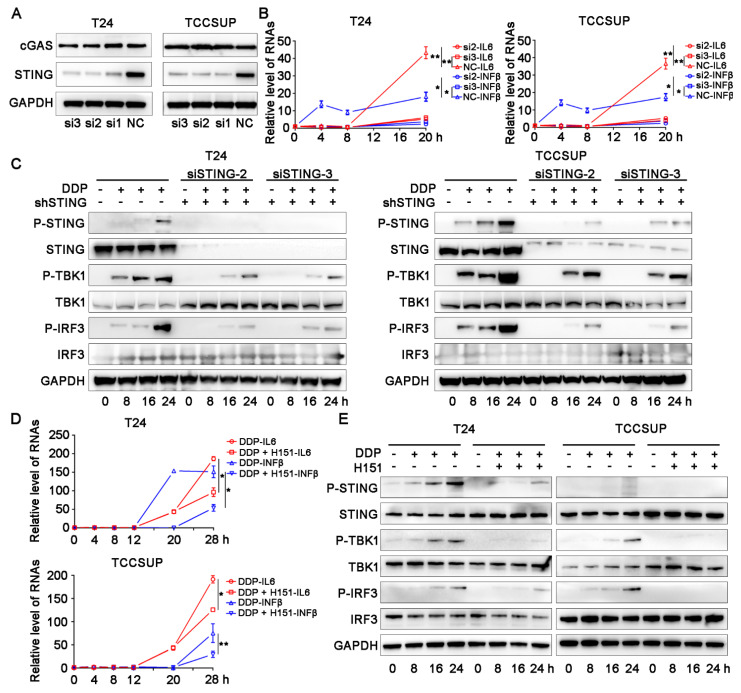
STING targeting siRNAs and STING specific inhibitor H151 significantly reversed cisplatin related immune effects. (**A**) Knockdown efficiency validation of STING in T24 and TCCSUP cell lines. GAPDH was used as control protein. (**B**) T24 and TCCSUP cell lines were treated with non-targeting (Scramble, Scr) or two STING-targeting siRNA (si2 and si3) for 48 h before treatment with 2 μg/mL cisplatin for 0 h, 4 h, 8 h, 12 h, 16 h, 20 h, respectively. IFN-β mRNA and IL-6 mRNA were then analyzed by qRT-PCR. (**C**) Western blotting results of key proteins involved in cGAS-STING pathway in T24 and TCCSUP cell lines treated with non-targeting (Scramble, Scr) or two STING-targeting siRNA (si2 and si3) for 48 h before treatment with 2 μg/mL cisplatin for 0 h, 8 h, 16 h, 24 h, respectively. GAPDH was used as control protein. (**D**) T24 and TCCSUP cell lines were co-treated with DMSO or H151 and 2 μg/mL cisplatin for 0 h, 4 h, 8 h, 12 h, 20 h, 28 h, respectively. IFN-β mRNA and IL-6 mRNA were then analyzed by qRT-PCR. (**E**) Western blotting results of key proteins involved in cGAS-STING pathway T24 and TCCSUP cell lines co-treated with DMSO or H151 and 2 μg/mL cisplatin for 0 h, 8 h, 16 h, 24 h, respectively. GAPDH was used as control protein. * *p* < 0.05, ** *p* < 0.01.

Expression of the STING protein is the basic element for the functional STING pathway. We first explored the basic transcription level of STING in bladder cancer cell lines in the CCLE database (https://portals.broadinstitute.org/ccle/about/, accessed on 11 October 2020) and found that the transcription level of STING was relatively abundant in bladder cancer, including T24 and another MIBC cell line TCCSUP (Figure 2A). ELISA and IFN-β reporter gene assays showed that the levels of IL-6 and IFN-β, which are activated downstream of the STING pathway, were significantly upregulated with cisplatin treatment in the T24 and TCCSUP cells (Figure 2B,C). qPCR confirmed that the transcription levels of cytokines, including IL-6, IFN-β, and TNF-α, were significantly upregulated after cisplatin treatment (Figure 2D). We also observed the translocation of p65 from the cytoplasm to the nucleus (Figure 2E). The canonical activation of the STING pathway is mainly based on the phosphorylation of STING (Ser366), TBK (Ser173), and IRF3 (Ser396) [25,26,27,28]. We then performed western blotting assays and found that cisplatin treatment significantly upregulated the cGAS-STING pathway accompanied by a significant increase in the DNA damage marker γ-H2A.X in the two cell lines compared with their corresponding control groups (Figure 2F). In the rescue assay, the use of two STING-targeting siRNAs (Figure 3A–C) and the STING-specific inhibitor H151 (Figure 3D,E) significantly reversed cisplatin-induced IL-6 and IFN-β levels compared with their control groups. H151 specifically targets the transmembrane cysteine residue 91 of STING and thereby blocks its palmitoylation which is indispensable for the recruitment of downstream signaling factors [29]. These results suggested that the cGAS-STING pathway was deeply involved in the cisplatin-related immune response.

### 3.3. Modulation of STING Did Not Affect the Proliferation of Bladder Cancer In Vitro

Since cisplatin activates cGAS-STING signaling in bladder cancer cell lines, the role of cGAS-STING in bladder cancer progression remains unclear. The CCK-8 cell proliferation assay (Figure S1C) and clonal formation assay (Figure S1D,E) indicated the absence of significant basic proliferation bias between the STING knockdown groups and the control groups in the two cell lines. We found that the levels of STING in patients from the TCGA bladder cancer cohort were not significantly associated with the overall survival rate (OS) or disease-free survival rate (DFS) (Figure S1F,G). This finding implies that the activation states and expression levels of genes in the cGAS-STING pathway are not inevitably relevant.

### 3.4. Activation of cGAS-STING Suppressed Bladder Cancer in Cisplatin-Treated C57 Mice

**Figure 4:**
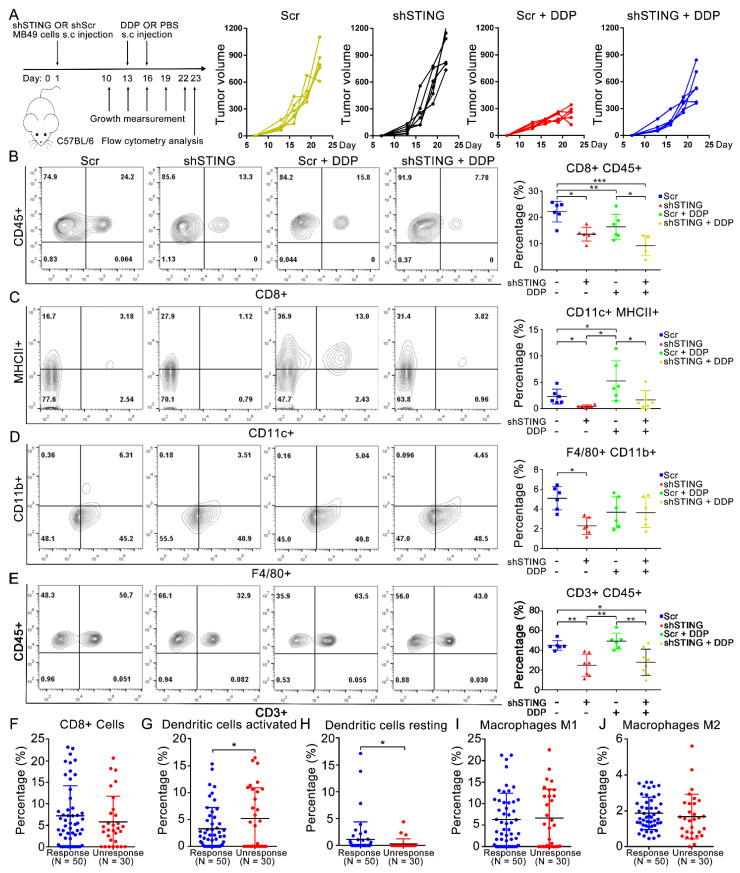
Activation of cGAS-STING suppressed bladder cancer in cisplatin-treated C57 mice. (**A**) Establish of bladder cancer transplantation models in C57 mice, left. Tumor volumes of different groups, right. (**B**–**E**) Infiltration percentages of (**B**) CD8+, CD45+ T cells, (**C**) CD11b+, MHCⅡ+ DCs, (**D**) F4/80+, CD11c+ Macrophage, (**E**) CD3+, CD45+ cells in MB49 transplantation tumors. (**F**–**J**) CYBERSORT predicted infiltration percentages of CD8+ T cells (**F**), DCs activated cells (**G**), DCs resting cells (**H**), Macrophages M1 cells (**I**), and Macrophages M2 cells (**J**) in response group (*n* = 50, CR or PR) was significantly higher than non-response group (*n* = 30, PD or SD) after cisplatin-based chemotherapy. * *p* < 0.05, ** *p* < 0.01, *** *p* < 0.001. DDP, cisplatin.

To explore the phenotype in vivo, we constructed an MB49 shSTING cell line and an MB49 scramble control cell line (mouse-derived) by lentivirus transfection (Figure S1H). To exclude the possible effects caused by different cell proliferation rates and cisplatin sensitivity between the two constructed cell lines, we verified that there was no significant difference in cell proliferation (Figure S1I,K,L) or basic IC50 of cisplatin treatment between the two groups (Figure S1J). At the same time, we noticed that the cisplatin treatment activated the cGAS-STING signal in the MB49 scramble control group but not the MB49 STING knockdown groups (Figure S1M). We then established a subcutaneous tumor transplantation model in C57 mice and injected cisplatin or PBS subcutaneously on days 13 and 16 (Figure 4A left). The tumors were harvested on day 23, and the results showed that the tumor volumes in the cisplatin-treated group were significantly reduced compared with those in the corresponding control group (Figure 4A right). Compared with the STING knockdown group, the tumor volume and relative tumor weight of the STING WT group were significantly reduced after cisplatin treatment (Figure S2A–C).

### 3.5. Activation of cGAS-STING Results in CD8+ T Cell and DC Infiltration in Cisplatin-Treated Tumors in a Transplantation Model

To explore whether the difference in MB49-derived tumors is related to immunity, we analyzed tumor-associated infiltrating immune cells by flow cytometry. With cisplatin treatment, we found that compared with the STING knockdown group, the percentage of CD8+ CD45+ CD11c+ MHCII+ (dendritic cells, DCs), and CD3+ CD45+ cells were significantly higher in the STING WT group (Figure 4B,C,E) while the percentage of F4/80+, CD11b+ (macrophage) infiltration was not significantly different (Figure 4D). Compared with the control group, the percentage of CD8+ CD45+, CD3+ CD45+, and F4/80+ CD11b+ cells in the cisplatin-treated groups decreased significantly while the percentage of CD11c+ MHCII+ infiltrating cells was significantly increased. We also used CIBERSORT (https://cibersort.stanford.edu/, accessed on 5 November 2020) [30] to analyze the percentage of immune cell infiltration in bladder cancer patients who received preoperative cisplatin chemotherapy in the TCGA bladder cancer cohort (Figure 4F–J). Consistent with our vivo experimental results, the resting dendritic cells infiltrated in the cisplatin-responsive group (*n* = 50, CR or PR) was significantly higher than that in the non-responder group (*n* = 30, PD or SD), while the infiltration of activated dendritic cells showed the opposite result. Studies have reported that CCL20 and CXCL14 are important chemokines in DC cells. We found that the transcription levels of CCL20 and CXCL14 in T24 and TCCSUP cells were significantly increased in the cisplatin treatment groups (Figure S2D,E), and the correlation analysis showed that CCL20 and CXCL14 were significantly correlated with the cGAS-STING signal in the TCGA bladder cancer cohort (Figure S2F). This finding implies that DCs play a unique role in the bladder cancer microenvironment after cisplatin treatment [31]. However, the specific function of cisplatin-induced DCs in the bladder cancer microenvironment needs to be further explored.

### 3.6. Cisplatin-Induced dsDNA May Play an Important Role in Enhancing STING Signaling

**Figure 5:**
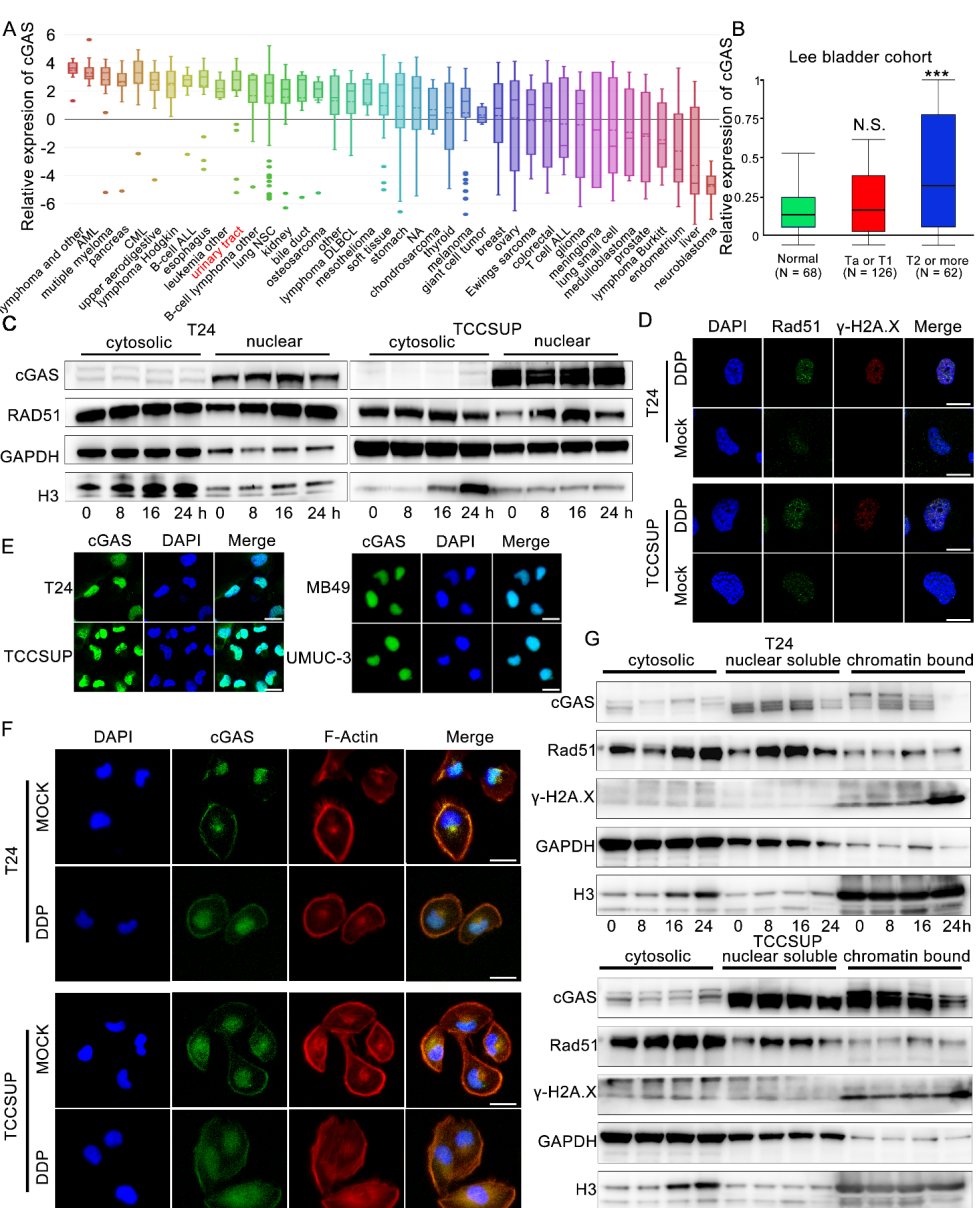
Cisplatin induced dsDNA and the releasing of chromatin bound cGAS may function as an important role in enhancing STING signal. (**A**) Basic transcription levels of cGAS in bladder cancer cell line on CCLE database. (**B**) Relative transcriptional expression of cGAS in different T stages (Normal N = 68, Ta or T1 N = 126, and T2 or more N = 62) in Lee bladder cohort. (**C**) Western blotting of subcellular distribution of cGAS, Rad51 GAPDH, and Histone H3 proteins. GAPDH was used as cytosolic control protein, and histone H3 was used as nuclear control proteins. T24 and TCCSUP cell treated with 2 μg/mL cisplatin or PBS at 0 h, 8 h, 16 h, 24 h time points. (**D**) Immunofluorescence of expression and distribution of Rad51 and γ-H2A.X proteins in T24 and TCCSUP cell lines treated with μg/mL cisplatin or PBS for 24 h. DDP, cisplatin. Scale bars represent 10 μm. (**E**) Immunofluorescence of cGAS subcellular distribution of T24, TCCSUP, MB49 and UMUC-3 cell lines. Scale bars represent 5 μm. (**F**) Immunofluorescence of cGAS subcellular distribution of T24 and TCCSUP cell lines treated with 2 μg/mL cisplatin or PBS. Scale bars represent 5 μm. (**G**) Western blotting of subcellular distribution of cGAS, Rad51, γ-H2A.X, GAPDH and Histone H3 proteins. GAPDH was used as cytosolic and nuclear soluble control protein, and Histone H3 was used as chromatin bound control proteins. T24 and TCCSUP cells were treated with 2 μg/mL cisplatin or PBS at 0 h, 8 h, 16 h, 24 h time points. DDP, cisplatin, N.S., *p* > 0.05, *** *p* < 0.001.

As an important protein that can recognize dsDNA and catalyze the production of 2,3-cGAMPs, the expression of the cGAS protein is the most important upstream of STING activation. We then explored the basic transcription level of cGAS in bladder cancer cell lines in the CCLE database (https://portals.broadinstitute.org/ccle/about, accessed on 11 October 2020) and confirmed the high transcription level of cGAS in bladder cancer cell lines (Figure 5A, rank 11th among 40 tumor types). T24 and TCCSUP also showed relatively high transcription levels of cGAS (Figure S2G), and we also noted that MIBC cells had significantly increased cGAS expression levels in the Oncoming Database (Figure 5B). Cisplatin has been reported to induce dsDNA fragments through the accumulation of DNA damage and inhibition of the DNA homologous complementary repair pathway [17]. We observed a significant increase in the DNA damage marker γ-H2A.X in T24 and TCCSUP cell lines treated with cisplatin (Figure 2F). Rad51 is an important protein involved in homologous complementary repair of DNA, and the upregulation and assembly of nuclear Rad51 is also the classical marker of increased DNA damage [32]. We found that nuclear Rad51 increased and was significantly accumulated after cisplatin stimulation while the cytosolic Rad51 levels were not changed (Figure 5C,D). When dsDNA induced by DNA damage leaks into the cytoplasm, histone H3 bound to dsDNA will be brought into the cytoplasm at the same time, resulting in an increased level of cytosolic H3 protein [33]. Consistent with our results, we observed that the level of H3 in the cytoplasm increased significantly as the time of cisplatin stimulation increased (Figure 5C), which also suggested that dsDNA might leak from the nucleus continuously after cisplatin treatment. Furthermore, we found that cisplatin also induced micronuclei in the T24 and TCCSUP cell lines (Figure S2H). These are important factors that activate the cGAS-STING pathway.

### 3.7. The Release of Chromatin-Bound cGAS Is Important to Activate Downstream STING

We tried to explore the role of cGAS in cisplatin-related STING activation and found that the expression level of total cGAS did not change significantly after cisplatin stimulation in the two cell lines (Figure 2E). The subcellular distribution of cGAS is closely related to its function [18,19,20,21,22]. First, we confirmed that the cGAS protein of T24 and TCCSUP cell lines was mainly located in the nucleus (Figure 5C,E). The same results were also shown in the MB49 and UMUC3 cell lines (Figure 5E). Then, we explored whether the subcellular distribution of cGAS was changed after cisplatin treatment. Although the morphology of T24 and TCCSUP cells became enlarged after cisplatin stimulation, the distribution of cGAS did not change significantly (Figure 5F). The western blotting results of nuclear-cytoplasmic separation also showed that cGAS distribution in the cytoplasm and nucleus did not change significantly in the two cell lines (Figure 5C). Recently, many scholars have found that cGAS can bind with histones H2 and H3 on the nucleosomes to form stable structures and prevent cGAS from binding with dsDNA [18,19,20,21,22]. Based on these findings, we performed a subcellular component western blotting assay and found that most cGAS was bound to the nucleosome in the nucleus. After cisplatin stimulation, especially at 24 h, the binding of cGAS in the nucleosomes in the two cell lines was significantly reduced while γ-H2A.X was significantly increased compared with their corresponding control groups (Figure 5G). Thus, free cGAS might reacquire the ability to bind to dsDNA and then activate downstream STING-dependent immune signals.

## 4. Discussion

In this work, we reported that cisplatin treatment of bladder cancer cells produced specific immune effects, which mainly included the secretion of important cytokines (such as interferon type I cytokines, IFN-β, and IL6). Interestingly, some studies showed that gemcitabine, another common chemotherapy drug, also had antitumor immune effects by stimulating interferon-γ expression and recruiting related immune cells [34,35,36]. These specific immune effects may provide novel explanation for the satisfying pathologic downstaging results of neoadjuvant therapy combined immunotherapy in MIBC [37]. Unfortunately, keynote 361 trial exhibited that the combination of platinum-based chemotherapy and pembrolizumab did not provide significant survival advantages as compared to platinum-based chemotherapy alone [38]. The increased PD-L1 expression induced by chemotherapy [6,7,39] and timing for introducing immunotherapy in the treatment of advanced urothelial carcinoma might explain the negative result, because JAVELIN bladder 100 trial found that avelumab maintenance treatment possessed huge survival benefits for patients responding to chemotherapy [40]. Therefore, more efforts should be paid to explore the mechanism and application of combination therapy.

The expression levels of STING and other components involved in the STING pathway were not associated with OS and DFS in bladder cancer patients. However, in a mouse tumor model, we confirmed that STING knockdown in bladder cells had no significant effect on tumor volume and weight while STING-knockdown combined with cisplatin treatment led to significant increases in tumor volume and weight compared with the STING-WT combined with cisplatin treatment. These results suggest that cisplatin-related STING-dependent signaling strongly inhibits bladder cancer progression, which is consistent with many previous studies [6,7,39,41]. These differences might be explained by the higher infiltration levels of CD8+ T cells and DCs in the STING-WT combined with cisplatin group than the STING-knockdown combined with cisplatin group. The infiltration levels of CD8+ T cells in tumor tissues were recently proven to be consistent with the functional cGAS-STING pathway [42]. The infiltration and activation of DCs in tumor tissues is closely related to specific chemokines in the tumor microenvironment. Chemokines, such as CCL20 and CXCL14, can recruit DCs to tumor tissue to inhibit tumor proliferation and metastasis [43,44]. In our experiment, we also observed increased transcription levels of CCL20 and CXCL14 in the T24 and TCCSUP cell lines after cisplatin treatment. It has also been reported that intrinsic cGAMPs in tumor cells can be transferred to DCs through gap junctions and activate the immune response of DC cells [45,46]. Thus, DCs may play an important role in cisplatin-related cGAS-STING immune effects in bladder cancer, although their underlying mechanism and specific functions in the tumor microenvironment remain to be clarified.

Urinary tract tumors, including bladder cancer, show relatively high expression of cGAS. Studies have shown that tumor cells with high expression of the cGAS protein have relatively high cGAS-STING pathway activity [33], because tumor cells often have higher levels of intracellular dsDNA due to more frequent DNA damage and repair cycles, which can activate cGAS and generate abundant cGAMPs, which ultimately activates STING downstream [45,46]. As a classical DNA crosslinking agent, cisplatin can strongly induce DNA damage in multiple tumor types. DNA damage leads to dsDNA leakage from the nucleus to the cytoplasm [4,5,33]. We observed that with the time of cisplatin induction, the level of histone H3 in the cytoplasm increased gradually, which indicated that dsDNA was constantly leaking from the nucleus [33]. At the same time, we observed the production of micronuclei in the cisplatin-treated groups. Micronuclei were reported not only as a marker of DNA damage but also as a factor for activating the STING signal [5].

The functional state of cGAS is closely related to its subcellular localization. For instance, translocation of cGAS from the cytoplasm to the cell membrane helps THP-1 and HeLa cells recognize the exogenous virus DNA [47]. Nuclear translocation of cGAS can affect its functions by suppressing homologous recombination-mediated repair [17]. However, we found that cGAS was mainly located in the nucleus in the T24 and TCCSUP cell lines, and this subcellular distribution was not changed after cisplatin treatment. Studies have shown that the binding capacity of cGAS to nucleosomes is much greater than that of cGAS to dsDNA [18,19,20,21,22,48]. Based on this important finding, many research groups have further discovered that cGAS can bind with histones H2 and H3 on nucleosomes to form stable structures and prevent cGAS from binding with dsDNA [18,19,20,21,22]. Therefore, we then explored the state of chromatin binding cGAS after cisplatin treatment and found that with prolonged cisplatin treatment, the DNA damage marker γ-H2A.X increased significantly while chromatin-bound cGAS decreased significantly. In multiple tumor types, the activation of cGAS is thought to inhibit tumor proliferation by activating the STING-dependent immune response, although this function is weakened due to the binding of nucleosomes [6,7,39]. These results indicate that cisplatin may hinder the stability of nucleosomes by distorting the natural structure of DNA [49,50]. Free cGAS may reacquire the ability to bind to dsDNA and then activate downstream STING-dependent immune signals. However, whether dissociated cGAS is transferred to the cytoplasm and further activates STING still needs to be proven.

In summary, our findings indicated a cisplatin dependent cGAS-STING signal in bladder cancer. This signal could be enhanced by accumulation of dsDNA and chromatin dissociated cGAS and would finally recruit infiltration DCs and CD8+ T cells in bladder cancer tumor microenvironment. However, the specific role and effect of this signal towards the bladder cancer tumor microenvironment needs to be further clarified.

## Data Availability

The RNA-seq data generated in this study is available in GEO under accession number GSE165767. Other data that supports the findings of this study are available from the corresponding author upon request.

## Supplementary Materials

The following supporting information can be downloaded at: https://www.mdpi.com/article/10.3390/cells11193011/s1. Figure S1. Expression of STING did not affect proliferation of T24, TCCSUP, and MB49 cell lines in vitro; Figure S2. Expression of STING did not affect proliferation and cisplatin sensitivity of MB49 cell lines; Table S1. The sequences of msSTING and siSTING and specific primers; Table S2. The sequencing coverage and quality statistics for each sample.
