# Supplementary material for: Activation of cGAS-STING Signal to Inhibit the Proliferation of Bladder Cancer: The Immune Effect of Cisplatin
Source: Cells. 2022 Sep 27;11(19):3011. doi: 10.3390/cells11193011 (PMC9564335; doi:10.3390/cells11193011)
Supplement: Supplementary file 1 [file cells-11-03011-s001.zip › cells-1894019-supplementary.pdf]

Supplementary Materials

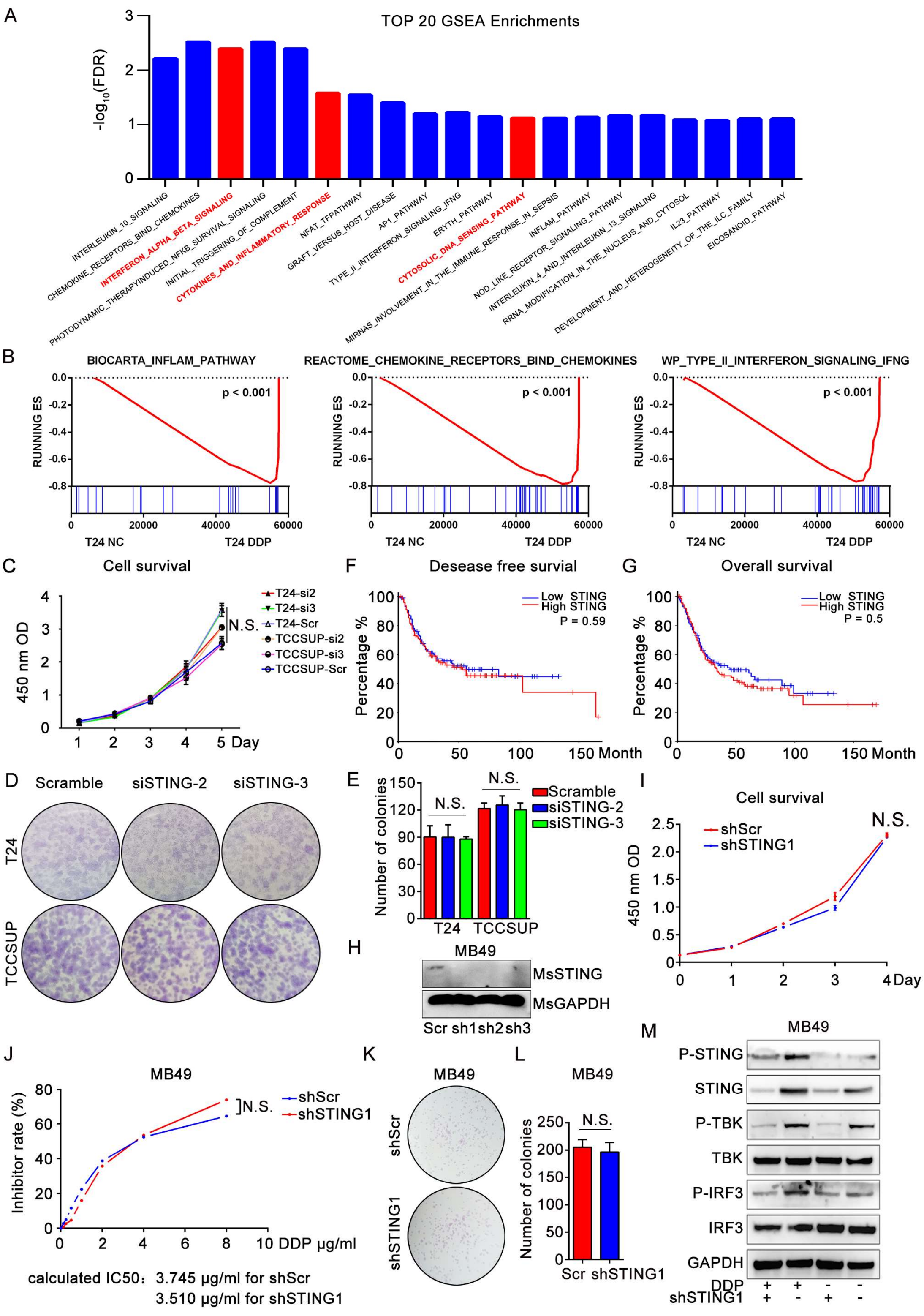

Figure S1. Expression of STING did not affect proliferation of T24, TCCSUP and MB49 cell lines in vitro. (A) Top 20 GSEA enrichment pathways in T24 cell lines with cisplatin treatment, 2 μg/ml for 24 h. (B) Inflam- and immune- relative enrichment pathway in T24 cell lines with cisplatin treatment, 2 μg/ml for 24 h. (C) T24 and TCCSUP cell lines were treated with non-targeting (Scramble, Scr) or two STING-targeting siRNA (si2 and si3) for 48 h before adding 1640 medium containing

10% CCK-8 regent. The 450 mm OD of each groups were measure at day 1, 2, 3, 4, 5. (D, E) Clonal formation assay of T24 and TCCSUP cell lines treated with non-targeting (Scramble, Scr) or two STING-targeting siRNA (si2 and si3) for 48 h. (F, G) Kaplan-Meier analysis of (F) overall survival rates and (G) disease-free survival rates stratified by low STING expression (n = 201) and high STING expression (n = 201) from The Cancer Genome Atlas (TCGA) database. The median was used as the dividing line, and STING upregulation showed none significantly associated with overall and disease-free survival rates. DDP, cisplatin. (H) Knockdown efficiency validation of shSTING lentivirus in MB49 cell lines. GAPDH was used as control protein. (I) MB49 cell lines were treated with non-targeting (Scramble, Scr) lentivirus or STING-targeting lentivirus for 48 h before adding 1640 medium containing 10% CCK-8 regent. The 450 mm OD of each groups were measure at day 0, 1, 2, 3, 4. (J) Calculated cisplatin IC50 of MB49 cell lines treated with non-targeting (Scramble, Scr) lentivirus or STING-targeting lentivirus for 48 h. (K, L) Clonal formation assay of MB49 cell lines treated with non-targeting (Scramble, Scr) lentivirus or STING-targeting lentivirus for 48 h. (M) Western blotting results of key proteins involved in cGAS-STING pathway in MB49 cell lines treated with 2 μg/ml cisplatin or PBS for 24 h combined with or without STING-targeting treatment. GAPDH was used as the control protein. DDP, cisplatin. N.S., P > 0.05.

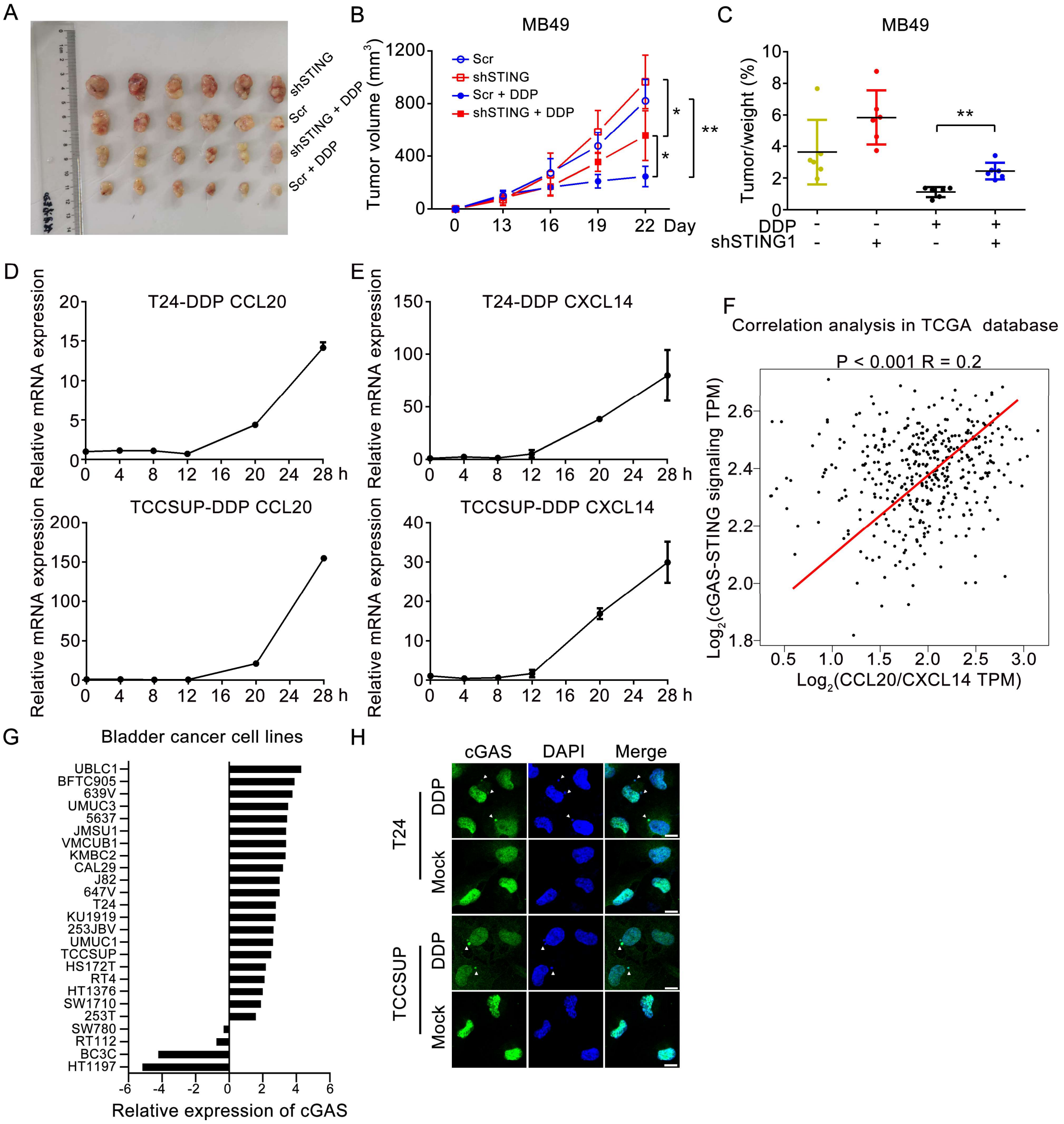

Figure S2. Expression of STING did not affect proliferation and cisplatin sensitivity of MB49 cell lines. (A) Different groups of tumor volumes in day 23. (B) Different groups of tumor proliferation curves. (C) Relative tumor weights of different groups. *P < 0.05, **P < 0.01. (D, E) T24 and TCCSUP cell lines were treated with 2 μg/ml cisplatin for 0 h, 4 h, 8 h, 12 h, 20 h, 28 h, respectively. CCL20

(D) and CXCL14 (E) mRNA were then analyzed by qRT-PCR. (F) Correlation analysis between cGAS-STING signaling and CCL20/CXCL14 expression in TCGA database. $P < 0.001$, $R = 0.2$. (G) Basic transcription levels of cGAS in bladder cancer cell line on CCLE database. (H) Cisplatin (2 μg/ml cisplatin for 24 h) induced micronuclei in T24 and TCCSUP cell lines. Scale bar represent 5 μm.

Table S1. The sequences of msSTING and siSTING and specific primers.

shRNA	Sequences
MsSTING-1-Forward	CCGGAGAGGTCACCGCTCCAAATATCTCGAGATATTTGGAGCGGTGACCTCTTTTTG
MsSTING-1-Reverse	AATTCAAAAAGAGGTCACCGCTCCAAATATCTCGAGATATTTGGAGCGGTGACCTCT
MsSTING-2-Forward	CCGGATGATTCTACTATCGTCTTATCTCGAGATAAGACGATAGTAGAATCATTTTTG
MsSTING-2-Reverse	AATTCAAAAAATGATTCTACTATCGTCTTATCTCGAGATAAGACGATAGTAGAATCAT
MsSTING-3-Forward	CCGGCAACATTCGATTCCGAGATATCTCGAGATATCTCGGAATCGAATGTTGTTTTTG
MsSTING-3-Reverse	AATTCAAAAACAACATTCGATTCCGAGATATCTCGAGATATCTCGGAATCGAATGTTG
siRNA	Sequences
siSTING-1	CATGGTCATATTACATCGGAT
siSTING-2	GCTGGCATGGTCATATTACAT
siSTING-3	GTCCAGGACTTGACATCTTAA
PCR Primer	Sequences
CXCL1-Forward	ACAGTGTGTGGTCAACATTTCT
CXCL1-Reverse	AGCCCCTTTGTTCTAAGCCA
CXCL2-Forward	CAGTGTGTGGTCAACATTTCTCA
CXCL2-Reverse	TGCTCTAACACAGAGGGAAACA
CXCL3-Forward	CGCCCAAACCGAAGTCATAG
CXCL3-Reverse	TCTGGTAAGGGCAGGGACC
CXCL4-Forward	CCACACTTAACGGAGAGCCT
CXCL4-Reverse	AGGTGGTCTTCACACACAGG
CXCL5-Forward	TGCTGAGCTTTTTAGATGCCT
CXCL5-Reverse	AGACTATGAACCAATGAGACACA
CXCL6-Forward	ACGCTGAGAGTAAACCCCAA
CXCL6-Reverse	CCAGACAAACTTGCTTCCCG
CXCL7-Forward	GTGATCGGGAAAGGAACCCA
CXCL7-Reverse	AGCAGATTCATCACCTGCCA
CXCL8-Forward	TGGACCCCAAGGAAAACTGG
CXCL8-Reverse	AAGTTTCACTGGCATCTTCACTG
CXCL14-Forward	ATGAAGCCAAAGTACCCGCA
CXCL14-Reverse	CTTCTCGTTCCAGGCGTTGT
CXCL16-Forward	GGCACCTGACTCTAATACCTGA
CXCL16-Reverse	GCAGTGGCTGGTTAGTCCTAT
CCL5-Forward	CAGTCGTCCACAGGTCAAGG
CCL5-Reverse	TTCTCTGGGTTGGCACACAC
CCL19-Forward	CCATCCCTGGGTACATCGTG
CCL19-Reverse	GCAGTCTCTGGATGATGCGT
CCL20-Forward	AACCATGTGCTGTACCAAGAGT
CCL20-Reverse	AAGTTGCTTGCTTCTGATTCGC
CCL21-Forward	TGCAGCATCTGGACAAGACA
CCL21-Reverse	CCTTTAGGGGTCTGTGACCG
IL-6-Forward	CAGCCCTGAGAAAGGAGACAT
IL-6-Reverse	GGTTCAGGTTGTTTTCTGCCA
TNF-α-Forward	CCCGAGTGACAAGCCTGTAG
TNF-α-Reverse	TGAGGTACAGGCCCTCTGAT
INF-β-Forward	CAGCATCTGCTGGTTGAAGA
INF-β-Reverse	CATTACCTGAAGGCCAAGGA

Table S2. The sequencing coverage and quality statistics for each sample.

Sample ID	Total number of sequenced reads	Total number of uniquely mapped non-duplicate reads[a]	RNA integrity number (RIN)	Ratio of all reads aligned to rRNA regions to total uniquely mapped reads (rRNA rate)	Ratio of exon-mapped reads to total uniquely mapped reads (Expression Profile Efficiency)	Total number of detected transcripts with reads ≥ 1[b]
T24DDP2	43688566	41149832(94.19%)	>7	unknown	5765554923(91.2064%)	24988
T24DDP1	43426958	40877691(94.13%)	>7	unknown	5757569876(91.6351%)	24731
T24DDP3	41373478	38932654(94.1%)	>7	unknown	5384577085(90.0616%)	25180
T24NC2	46041354	43248261(93.93%)	>7	unknown	6152975627(92.4967%)	23686
T24NC3	43789786	41206904(94.1%)	>7	unknown	5889299096(92.9955%)	23167
T24NC1	46520052	43510283(93.53%)	>7	unknown	6229786100(93.1316%)	23425
